# Malignancy is increased in patients with antineutrophil cytoplasmic antibody-associated vasculitis in China

**DOI:** 10.1186/s13075-024-03345-2

**Published:** 2024-05-31

**Authors:** Xiang-Yu Han, Zhi-Ying Li, Ming-Hui Zhao, Mark A. Little, Min Chen

**Affiliations:** 1 Renal Division, Department of Medicine, Peking University First Hospital, Peking University Institute of Nephrology, Key Laboratory of Renal Disease, Ministry of Health of China, Key Laboratory of Chronic Kidney Disease Prevention and Treatment (Peking University), Ministry of Education, Research Units of Diagnosis and Treatment of Immune-mediated Kidney Diseases, Chinese Academy of Medical Sciences, Beijing, 100034 China; 2 Trinity Kidney Centre, Trinity Translational Medicine Institute, Trinity College Dublin, Irish Centre for Vascular Biology, Trinity College Dublin, Dublin, Ireland

**Keywords:** ANCA, Vasculitis, Malignancy, Cyclophosphamide

## Abstract

**Objective:**

It has been reported that in western countries malignancy risk was higher in patients with antineutrophil cytoplasmic antibody (ANCA)-associated vasculitis (AAV) compared with that in the general population. In the current study, we investigated the incidence, spectrum and risk factors of malignancy in Chinese AAV patients.

**Methods:**

AAV patients diagnosed from 1995 to 2021 in Peking University First Hospital with a follow-up more than 12 months were recruited. Standardized incidence ratios (SIR) were calculated to describe the risk of malignancy, adjusted for sex, age and follow-up time.

**Results:**

A total of 552 AAV patients were recruited, among which 23 patients had malignancies either preceding or concurrent with AAV diagnosis, and 43 of the remaining 529 patients developed malignancies within 4.3 ± 4.2 years post AAV diagnosis (SIR: 2.24; 95% CI: 1.68–2.99; *p* < 0.001). Among these 66 patients, twenty different sites of malignancy were observed, lung cancer being most frequent. To get exactly expected malignancies for the calculation of SIR, 529 patients without preceding or concurrent malignancies were included in the following analysis. Lung cancer was still the leading malignancy diagnosis (SIR: 5.01; 95% CI: 3.29–7.62), followed by malignancies in the kidney, bladder, ureter and prostate. Male gender (HR:2.84; 95%CI:1.36–5.96; *p* = 0.006) and older age (per year, HR:1.04; 95%CI:1.00-1.07; *p* = 0.038) were significantly associated with increased risk of malignancy. For patients with malignancy developed beyond 5 years after the diagnosis of AAV, a significantly higher malignancy risk was observed in those with a cumulative cyclophosphamide dose over 20.0 g (SIR: 11.54; 95% CI: 4.77–27.93; *p* < 0.001). Within the first 2 years after the diagnosis of AAV, the risk of malignancy was still significantly higher than that in the general population, but the cumulative cyclophosphamide dose was not significantly associated with malignancy occurrence in this subgroup of patients.

**Conclusions:**

Malignancy risk is higher in Chinese AAV patients than that in the general population, with a different malignancy spectrum from western countries. Both the use of cyclophosphamide and AAV *per se* might be associated with higher incidence of malignancy occurrence.

## Introduction

Antineutrophil cytoplasmic antibody (ANCA)-associated vasculitis (AAV) is a group of severe autoimmune diseases, including granulomatosis with polyangiitis (GPA), microscopic polyangiitis (MPA) and eosinophilic granulomatosis with polyangiitis (EGPA). AAV is characterized by the presence of circulating ANCA specific for myeloperoxidase (MPO) or proteinase 3 (PR3), and the involvement of small blood vessels, indicating the dysregulation of immunity [1, 2].

Without efficient treatment, AAV is usually a life-threatening disease, with a one-year mortality rate of ~ 80%. Immunosuppressive therapy has greatly improved the survival of AAV patients in the past decades [3, 4]. Thus, long-term complications and health-related quality of life received increasing attention. Increased malignancy incidence rate was observed in studies based on AAV patients from western countries. Regarding the risk factors, it was reported that patients with a cumulative cyclophosphamide dose over 36 g had an increased risk of malignancies [5–9]. Moreover, increased preceding or concurrent malignancies were also observed, suggesting an intrinsically higher malignancy risk beyond immunosuppressive therapy in AAV patients [10–13].

It has been recognized that the disease spectrum and serotypes of ANCA in Chinese AAV patients are different from those in western countries [14, 15]. For treatment strategy, less cyclophosphamide exposure was reported in Chinese AAV patients [15, 16]. Therefore, studies on malignancy in Chinese AAV patients were of great interest and necessary. In the current study, malignancy incidence and spectrum were described and the standardized incidence ratios (SIR) of malignancy were calculated in Chinese AAV patients. Potential risk factors of malignancy were also explored.

## Materials and methods

### Patients

AAV patients diagnosed in Peking University First Hospital from 1995 to 2021 and followed up for at least 12 months were retrospectively recruited. All patients met the 2012 Chapel Hill Consensus Conference of AAV [2]. Exclusion criteria were as follows: ANCA-negative patients; patients with EGPA (since EGPA is increasingly considered a distinct type of AAV with different manifestations and outcomes compared with GPA and MPA) [17]; patients with secondary vasculitis or comorbid renal diseases [2]. Clinical information was recorded at the time of presentation as well as during follow-up. Disease activity was measured using the Birmingham Vasculitis Activity Score (BVAS) [18].

This study was conducted in accordance with the Declaration of Helsinki and was approved by the Ethics Committee of Peking University First Hospital.

### The standardised incidence ratios (SIR)

The malignancy incidence rate in the general population was retrieved from Chinese National Central Cancer Registry (NCCR) to compute the expected malignancies, adjusting by age, gender and duration of follow-up [19]. The malignancy incidence rate was multiplied by the number of each category to get exactly expected malignancies. Then observed malignancies were divided by expected malignancies to calculate SIR. The 95% confidence intervals (95% CIs) of SIR were obtained by exact Poisson regression models [20, 21].

### Statistical analysis

All the data in our study were performed by SPSS software (version 27.0, IBM Corp, Armonk, NY). Continuous variables in normal distribution were displayed as mean ± standard deviation (SD), and those in skewed distribution were shown as median and interquartile range (IQR). Categorical variables were shown as number and percentage. Baseline data of patients with and without malignancies were compared using the student’s t-test for continuous variables and the chi-square test for categorical variables as appropriate. Univariate and multivariable Cox regression were performed to analyze malignancy risk factors. P value less than 0.05 was regarded as statistically significant.

## Results

### General data of AAV patients

A total of 552 patients were included, among which 243 were male and 309 were female, with an average age of 61.4 ± 13.1 years old at the time of diagnosis of AAV. Five hundred and two (90.9%) patients were MPO-ANCA positive while the other 50 (9.1%) patients were PR3-ANCA positive. The follow-up time of the 552 patients was 5.2 ± 4.1 years. The median cumulative cyclophosphamide dose was 5.0 (IQR: 1.0–8.0) g, and no significant difference between patients with and without malignancies was observed. Immunosuppressive agents for maintenance therapy mainly included azathioprine, rituximab and mycophenolate mofetil, among which azathioprine was most commonly used. In this cohort, 324 (58.7%) of the 552 patients used azathioprine for maintenance therapy and the median duration of azathioprine treatment was 35.0 (IQR: 19.8–63.3) months, without significant difference between patients with and without malignancies. More detailed general data of the 552 patients were shown in Table 1.

**Table 1:** General data of AAV patients

Variables	All AAV patients (*n* = 552)	Patients with preceding or concurrent malignancies (*n* = 23)	Patients without preceding or concurrent malignancies (*n* = 529)	
			Patients without malignancies (*n* = 486)	Patients with malignancies (*n* = 43)
Age (years), mean (SD)	61.4 (13.1)	67.9 (11.1)	60.8 (13.2)	64.8 (12.0)
Gender (male/female)	243/309	11/12	206/280	26/17
Follow-up (years), mean (SD)	5.2 (4.1)	5.1 (5.3)	5.3 (4.0)	4.8 (4.8)
MPO-ANCA/PR3-ANCA, n	502/50	22/1	441/45	39/4
Scr (µmol/L), mean (SD)	376.0 (299.4)	256.4 (198.2)	383.1 (307.7)	350.4 (220.2)
BVAS, median (IQR)	17 (14–21)	16 (14–20)	17 (14–21)	16 (12–20)
Clinical manifestations at diagnosis (n, (%))				
Renal involvement	549 (99.5)	23 (100.0)	483 (99.4)	43 (100.0)
Pulmonary involvement	346 (62.7)	10 (43.5)	307 (63.2)	29 (67.4)
Ear Nose Throat involvement	181 (32.8)	4 (17.4)	162 (33.0)	15 (34.9)
Immunosuppressent				
Rituximab, n (%)	30 (5.4)	1 (4.3)	26 (5.3)	3 (7.0)
Cyclophosphamide, n (%)	471 (85.3)	9 (39.1)	424 (87.2)	38 (88.4)
Cumulative cyclophosphamide dose (g), median (IQR)	5.0 (1.0–8.0)	3.6 (1.8–6.2)	4.9 (0.8–7.7)	6.0 (3.0-9.5)
Non-glucocorticoid agents for maintenance therapy				
Azathioprine, n (%)	324 (58.7)	10 (43.5)	290 (59.7)	24 (55.8)
Rituximab, n (%)	37 (6.7)	0 (0)	34 (7.0)	3 (7.0)
Mycophenolate mofetil, n (%)	54 (9.8)	1 (4.3)	49 (10.1)	4 (9.3)
Other agents, n (%)	95 (17.2)	2 (8.7)	86 (17.7)	7 (16.3)
None, n (%)	42 (7.6)	10 (43.5)	27 (5.6)	5 (11.6)

Abbreviations: AAV, anti-neutrophil cytoplasmic antibodies associated vasculitis; ANCA, anti-neutrophil cytoplasmic antibodies; BVAS, Birmingham Vasculitis Activity Score; eGFR, estimated glomerular filtration rate; IQR, interquartile range; MPO, myeloperoxidase; PR3, proteinase 3; Scr, serum creatinine; SD, standard deviation

### Malignancy in AAV patients

A total of 66 malignancies were observed in 66 of the 552 (12.0%) AAV patients, including 23 (4.2%) preceding or concurrent malignancies and 43 (7.8%) malignancies occurring after the diagnosis of AAV. The 23 patients who had preceding or concurrent malignancies had an age of 67.9 ± 11.1 years at the diagnosis of AAV and, of those, lung cancer and breast cancer were the two leading types, followed by malignancies in the stomach, bladder, thyroid, kidney, throat, colon, ureter and endometrium.

Since the time from the diagnosis of AAV to the occurrence of malignancy was a prerequisite for the calculation of expected malignancies and SIR, 529 out of 552 patients without preceding or concurrent malignancies were included in the following analysis. Among these 529 patients, 43 patients developed 43 malignancies within 4.3 ± 4.2 years after the diagnosis of AAV. The malignancies risk was 2.24-fold higher than the general population (95%CI: 1.68–2.99), adjusted for age, gender and follow-up time. Lung cancer was again the leading type of malignancy and was observed in 23 (53.4%) patients with a SIR of 5.01 (95%CI: 3.29–7.62). Besides, malignancies risk of the kidney (SIR: 8.00; 95%CI: 1.90-33.69; *p* < 0.001), bladder (SIR: 4.76; 95%CI: 1.16–19.62; *p* = 0.031), ureter (SIR: 45.46; 95%CI: 11.05-187.02; *p* < 0.001), renal pelvis (SIR: 27.78; 95%CI: 3.82-202.23; *p* = 0.001), testis (SIR: 175.44; 95%CI: 23.39-1264.63; *p* < 0.001), prostate (SIR: 4.65; 95%CI: 1.13–19.15; *p* = 0.033) and thymus (SIR: 19.23; 95%CI: 2.66-138.85; *p* = 0.003) in our AAV cohort were significantly higher than that in the general population (Table 2).

**Table 2:** SIR of different malignancy sites in AAV patients without preceding or concurrent malignancies (*n* = 529)

Sites	Observed malignancies	Expected malignancies	SIR (95%CI)	*P*
All sites	43	19.21	2.24 (1.68–2.99)	< 0.001
Lung	22	4.39	5.01 (3.29–7.62)	< 0.001
Kidney	2	0.25	8.00 (1.90-33.69)	< 0.001
Liver	2	1.73	1.16 (0.29–4.65)	0.838
Bladder	2	0.42	4.76 (1.16–19.62)	0.031
Ureter	2	0.044	45.46 (11.05-187.02)	< 0.001
Prostate	2	0.43	4.65 (1.13–19.15)	0.033
Stomach	2	2.26	0.89 (0.22–3.55)	0.863
Renal pelvis	1	0.036	27.78 (3.82-202.23)	0.001
Ovary	1	0.23	4.35 (0.59–32.17)	0.162
Cervix	1	0.42	2.38 (0.33–17.27)	0.391
Testis	1	0.0057	175.44 (23.39-1264.63)	< 0.001
Breast	1	1.18	0.85 (0.12–6.06)	0.869
Rectum	1	0.97	1.03 (0.14–7.38)	0.976
Lymphatic system	1	0.30	3.33 (0.35–31.99)	0.297
Thymus	1	0.052	19.23 (2.66-138.85)	0.003
Non-melanoma skin cancer	1	0.16	6.25 (0.83–47.04)	0.075

Abbreviations: SIR, standardized incidence ratios

Malignancies of the 529 patients were stratified by gender and age as well, presented in Table 3. Both male and female AAV patients had a significant higher SIR compared with the general population (2.49; 95%CI: 1.72–3.59; *p* < 0.001 and 1.94; 95%CI: 1.22–3.09; *p* = 0.005, respectively).

**Table 3:** SIR of malignancy stratified by gender and age at diagnosis (*n* = 529)

Age (years)	Observed malignancies	Expected malignancies	SIR (95%CI)	*P*
Male				
< 60	7	1.09	6.42 (3.10-13.32)	< 0.001
≥ 60	19	9.36	2.03 (1.33–3.11)	0.001
All	26	10.45	2.49 (1.72–3.59)	< 0.001
Female				
< 60	4	1.84	2.17 (0.82–5.75)	0.118
≥ 60	13	6.92	1.88 (1.11–3.19)	0.020
All	17	8.76	1.94 (1.22–3.09)	0.005

Abbreviations: SIR, standardized incidence ratios

### Risk factors of malignancy for AAV patients

Univariate and multivariate Cox regression were performed in the 529 AAV patients without preceding or concurrent malignancies to screen potential risk factors of malignancy. Univariate Cox regression showed older age and male gender were significantly associated with the increased risk of malignancy occurrence in AAV patients. In the multivariate Cox regression analysis, age (per year, HR:1.04; 95%CI: 1.00-1.07; *p* = 0.038) and male gender (HR:2.84; 95%CI: 1.36–5.96; *p* = 0.006) were still significantly associated with the increased risk of malignancies. No obvious association between the cumulative cyclophosphamide dose and malignancies occurrence was found in AAV patients, even adjusted by age and gender (Table 4).　There was also no significant association between the duration of azathioprine or the use of other maintenance therapy and malignancies.

**Table 4:** Cox analysis for malignancies

	Univariable		Multivariable	
Variables	HR (95%CI)	*P*	HR (95%CI)	*P*
Age (per year)	1.03 (1.00-1.06)	0.037	1.04 (1.00-1.07)	0.038
male vs. female	2.06 (1.11–3.82)	0.022	2.84 (1.36–5.96)	0.006
MPO-ANCA vs. PR3-ANCA	0.99 (0.36–2.83)	0.991	1.25 (0.27–5.87)	0.774
Immunosuppressant*				
Pred + CYC vs. Pred + RTX	1.10 (0.11–1.94)	0.294	-	-
Cumulative cyclophosphamide dose (g)	1.02 (0.99–1.04)	0.162	1.02 (0.99–1.05)	0.092

Abbreviations: ANCA, anti-neutrophil cytoplasmic antibodies; CI, confidence interval; CYC, cyclophosphamide; HR, hazard ratio; MPO, myeloperoxidase; PR3, proteinase 3; Pred, prednisone; RTX, rituximab

*The ‘Pred + CYC’ group was treated with prednisone combined with cyclophosphamide. The ‘Pred + RTX’ group was treated with prednisone combined with rituximab

### Association of cyclophosphamide and malignancy occurrence

In the 529 AAV patients, the median dose of cumulative cyclophosphamide was 6.0 (IQR: 3.0-9.5) g in those with malignancy and 4.9 (IQR: 0.8–7.7) g in those without malignancy (*p* = 0.208).

In fact, it would take a long period of time for immunosuppresants, in particular, cyclophosphamide to manifest a potential effect on the development of malignancy. Therefore, we arbitrarily performed additional analysis of malignancies developed beyond 5 years after the diagnosis of AAV to better evaluate the association between malignancy and cyclophosphamide. In our cohort, 230 of the 529 patients had a follow-up time more than 5 years, and 13 patients of them developed malignancies, with a median cumulative cyclophosphamide dose of 13.0 (IQR: 9.7–16.7) g, which was significantly higher than that of patients without malignancy (4.5 g; IQR: 0-8.9; *p* < 0.001). Interestingly, in the 13 patients with malignancies, only the risk of urologic malignancies including those in the kidney (SIR: 11.11; 95%CI: 2.59–47.61; *p* < 0.001), bladder (SIR: 6.67; 95%CI: 1.12–36.96; *p* = 0.037) and renal pelvis (SIR: 38.46; 95%CI: 5.24-282.24; *p* < 0.001) were significantly higher compared with the general population (Table 5).

**Table 5:** Malignancy developed beyond 5 years after the diagnosis of AAV (*n* = 230)

Sites	Observed malignancies	Expected malignancies	SIR (95%CI)	*P*
All sites	14	13.93	1.01 (0.60–1.67)	0.985
Lung	6	3.14	1.91 (0.86–4.24)	0.111
Kidney	2	0.18	11.11 (2.59–47.61)	< 0.001
Bladder	2	0.30	6.67 (1.12–36.96)	0.037
Renal pelvis	1	0.026	38.46 (5.24-282.24)	< 0.001
Prostate	1	0.30	3.33 (0.35–31.92)	0.296
Stomach	2	1.61	1.24 (0.31–4.98)	0.759

Abbreviations: SIR, standardized incidence ratios

To better evaluate the potential impact of cyclophosphamide on malignancy, these 230 patients were further stratified based on the cumulative cyclophosphamide doses, and SIR was calculated for each subgroup. As shown in Table 6, significantly higher malignancies risk compared with the general population was observed when the cumulative cyclophosphamide dose was 10–20 g (SIR:2.70; 95%CI: 1.07-6.85; *p* = 0.036); when the cumulative cyclophosphamide dose was more than 20.0 g, much higher SIR was observed (SIR:11.54; 95%CI: 4.77–27.93; *p* < 0.001). Nevertheless, compared with patients of the cumulative cyclophosphamide doses less than 10.0 g, the malignancy risk was significantly higher only in those with the cumulative cyclophosphamide dose more than 20.0 g (OR:16.17; 95%CI:2.67–97.83; *p* = 0.007) (Table 6).

**Table 6:** SIR and OR stratified according to cumulative cyclophosphamide doses

Cumulative cyclophosphamide doses (g)	Patients	Observed malignancies	SIR (95% CI)	*P*	OR	*P*
0	7	1	1.16 (0.19–7.22)	0.871	1 (reference)	
(0,10]	106	5	0.75 (0.32–1.78)	0.520		
(10,20]	32	4	2.70 (1.07–6.85)	0.036	2.31 (0.61–8.76)	0.383
> 20	6	3	11.54 (4.77–27.93)	< 0.001	16.17 (2.67–97.83)	0.007

Abbreviations: OR, odds ratio; SIR, standardized incidence ratios

### Potential association between malignancy risk and AAV *per se*

In the current study, 23 patients had preceding or concurrent malignancies. In such circumstance, the malignancy occurrence could hardly be attributed to immunosuppressants, in particular, cyclophosphamide. In order to better explore the potential association between malignancy risk and AAV *per se*, we analyzed the data of patients within 2 years of follow-up. This time-point was arbitrarily selected since malignancy occurring within 2 years after the diagnosis of AAV is not likely attributed to the use of cyclophosphamide.

Fifty hundred and twenty-nine patients without preceding or concurrent malignancies in our cohort were included in this analysis and the follow-up was truncated at 2 years. Nineteen patients were diagnosed with malignancies within 2 years after the diagnosis of AAV, with a crude SIR of 2.70 (95%CI: 1.73–4.23; *p* < 0.001) as compared with the general population. Lung cancer was still the leading one during this period. There was no significant difference in the cumulative cyclophosphamide dose between AAV patients with and without malignancies within the first 2 years after diagnosis of AAV.

## Discussion

It has been increasingly reported from western countries that there was a higher malignancy incidence rate in AAV patients, compared with the general population, especially non-melanoma skin cancer, hematologic malignancy and bladder cancer [22–26]. To our knowledge, this was the first large Chinese population-based study on malignancy occurrence in AAV patients. In this study, 66 events of malignancy were observed in 552 AAV patients. Compared with the general population, increased malignancy occurrence was observed in our cohort, with a total SIR of 2.24, which was in line with previous studies in western countries [7, 25]. Regarding the spectrum of malignancies in our cohort, lung cancer was the leading one, while skin cancer or hematological malignancy was rarely observed. The spectrum of malignancies in AAV patients in China seemed quite different from that in western countries. An especially lower rate of skin cancer was observed in our cohort compared with that in the western population. It was reported that higher risk of skin cancer might be attributable to the use of azathioprine [8, 27]. Nevertheless, no significant difference of the median duration of azathioprine treatment was observed between those with malignancy and without malignancy (32.0 (24.0-44.5) vs. 36.0 (19.5–64.5) months, *p* = 0.676) in our cohort. Compared with the western population, skin malignancy incidence in Chinese general population was much lower, less ultraviolet radiation or distinctive skin sensitivity might partly illustrate the low skin malignancy incidence of our cohort [19, 28]. Less cyclophosphamide exposure in Chinese patients might be another possible contributor for the rarity of hematological malignancy [15, 16].

Regarding the risk factors of malignancy, male and older patients were more susceptible. Previous studies found that cumulative cyclophosphamide dose over 36 g was associated with development of malignancies [5], but actually Chinese AAV patients were under less exposure to cyclophosphamide than patients in previous studies from western countries [15, 16, 29], suggesting that the use of cyclophosphamide could not fully explain the higher malignancy incidence in Chinese AAV patients [9]. In our study, to better evaluate the association between malignancy and cyclophosphamide, data about all malignancies and malignancies developed beyond 5 years after the diagnosis of AAV were analyzed separately. Though the cumulative cyclophosphamide dose was not significantly associated with over-all risk of malignancies, when restricting to patients with malignancy developed beyond 5 years after the diagnosis of AAV, urologic malignancies became the leading one, and significantly higher dose of cyclophosphamide was observed in patients with malignancy than those without. Moreover, among those AAV patients with cumulative cyclophosphamide dose less than 10 g, the risk of malignancy developed beyond 5 years after the diagnosis of AAV was comparable to the general population, and malignancies risk was significantly higher when the cumulative cyclophosphamide dose was 10 ~ 20 g and over 20 g than that in the general population; compared with patients with the cumulative dose of cyclophosphamide less than 10 g, the malignancy risk was significantly higher only in those with the cumulative dose of cyclophosphamide more than 20.0 g. These findings indicated that higher cumulative dose of cyclophosphamide was associated with higher risk of malignancy. Since cyclophosphamide is still widely used in less developed area, our findings make sense in clinical decision, i.e., regarding malignancy risk, a cumulative dose of cyclophosphamide less than 10 g is relatively safe, while over 20 g should be cautious.

In fact, in AAV patients, immunosuppressive therapy was not the only contributor to malignancy. Therefore, we arbitrarily extracted the data of patients within 2 years of follow-up after the diagnosis of AAV since malignancy occurred during this period could hardly be not likely attributed to immunosuppressive therapy, as described above. In our study, a 2.70-fold higher risk of malignancy compared with the general population was observed in AAV patients within 2 years after the diagnosis of AAV, and no significant difference was found in the cumulative cyclophosphamide dose between AAV patients with and without malignancies. These results suggested that the development of malignancies in this subgroup of patients might be associated with AAV *per se* rather than cyclophosphamide. However, nearly all new-diagnosed AAV patients underwent lung examinations including chest X-ray and CT scan. Thus, increased detection of malignancies (especially lung cancer) in AAV patients within 2 years after the diagnosis of AAV might be partly owing to surveillance bias beyond AAV *per se*. Since both AAV and malignancy affects mainly older people, the aging immune system, i.e., immunosenescence and immune dysfunction, might be a contributor. In the condition of immunosenescence and immune dysfunction of these patients, AAV is associated with the loss of immune tolerance, while the loss of immune surveillance contributes to malignancy [30]. Nevertheless, the underlying mechanism between malignancy and AAV remains to be further investigated.

Since this study was from a single center, there were several limitations. First, as our center is nephrology specialty, selection bias of disease spectrum occurs inevitably. Second, some covariates like dialysis and kidney transplant status in our cohort might affect the calculation of malignancy risk. Third, the relatively short follow-up of some patients might result in the underestimate of malignancy incidence rate. Study with multi-center cohort, larger sample size, and longer follow-up is further needed.

## Conclusion

In conclusion, malignancy risk is higher in Chinese AAV patients than that in the general population, with a different malignancy spectrum from western countries. Both AAV *per se* and the use of cyclophosphamide might be were associated with increasing occurrence of malignancies.

## Data Availability

No datasets were generated or analysed during the current study.

## Abbreviations

ANCA: Antineutrophil cytoplasmic antibody
AAV: Antineutrophil cytoplasmic antibody-associated vasculitis
SIR: Standardized incidence ratios
MPA: Microscopic polyangiitis
GPA: Granulomatosis with polyangiitis
EGPA: Eosinophilic granulomatosis with polyangiitis
PR3: Proteinase 3
MPO: Myeloperoxidase
ANCA-GN: ANCA-associated glomerulonephritis
ESRD: End-stage renal disease
eGFR: Estimated glomerular filtration rate
BVAS: Birmingham Vasculitis Activity Score
IQR: Interquartile range
SD: Mean ± standard deviation
CI: Confidence interval
Scr: Serum creatinine
OR: Odds ratio
HR: Hazard ratio
CYC: Cyclophosphamide
Pred: Prednisone
RTX: Rituximab
